# A nitty-gritty aspect of correlation and network inference from gene expression data

**DOI:** 10.1186/1745-6150-3-35

**Published:** 2008-08-20

**Authors:** Lev B Klebanov, Andrei Yu Yakovlev

**Affiliations:** 1 Department of Probability and Statistics, Charles University, Sokolovska 83, Praha-8, CZ-18675, Czech Republic; 2 Department of Biostatistics and Computational Biology, University of Rochester, 601 Elmwood Avenue, Box 630, Rochester, New York 14642, USA

## Abstract

**Background:**

All currently available methods of network/association inference from microarray gene expression measurements implicitly assume that such measurements represent the actual expression levels of different genes within each cell included in the biological sample under study. Contrary to this common belief, modern microarray technology produces signals aggregated over a random number of individual cells, a "nitty-gritty" aspect of such arrays, thereby causing a random effect that distorts the correlation structure of intra-cellular gene expression levels.

**Results:**

This paper provides a theoretical consideration of the random effect of signal aggregation and its implications for correlation analysis and network inference. An attempt is made to quantitatively assess the magnitude of this effect from real data. Some preliminary ideas are offered to mitigate the consequences of random signal aggregation in the analysis of gene expression data.

**Conclusion:**

Resulting from the summation of expression intensities over a random number of individual cells, the observed signals may not adequately reflect the true dependence structure of intra-cellular gene expression levels needed as a source of information for network reconstruction. Whether the reported effect is extrime or not, the important point, is to reconize and incorporate such signal source for proper inference. The usefulness of inference on genetic regulatory structures from microarray data depends critically on the ability of investigators to overcome this obstacle in a scientifically sound way.

**Reviewers:**

This article was reviewed by Byung Soo KIM, Jeanne Kowalski and Geoff McLachlan

## 1. Introduction

Inferring gene regulatory networks from microarray data has become a popular activity in recent years, resulting in an ever increasing volume of publications. There are many pitfalls in network analysis that remain either unnoticed or scantily understood. A critical discussion of such pitfalls is long overdue. In the present paper, we discuss one feature of microarray data the investigators need to be aware of when embarking on a study of putative associations between elements of networks and pathways. We believe that the present discussion pinpoints the crux of the difficulty in correlation analysis of microarray data and network inference based on correlation measures. The same caveat is of even greater concern in reference to more sophisticated methodologies that are designed to extract more information from the joint distributions of expression signals, Bayesian network inference being a relevant example. In a paper published in 2003, Chu et al. [1] pointed out the important fact that the measurements of mRNA abundance produced by microarray technology represent aggregated expression signals and, as such, may not adequately reflect the molecular events occurring within individual cells. To illustrate this conjecture, the authors proceeded from the observation that each gene expression measurement produced by a microarray is of the sum of the expression levels over many cells.

## 2. Aggregated expression intensities

Let *ν *be the number of cells contributing to the observed expression signal *U *(see Remark 1 below) and denote by *X*_*i *_the expression level of a given gene the expression level of a given gene in the *i*th cell. The notation *Y*_*i *_is used for the second gene in a given pair of genes. A simplistic model of the observed expression signals in this pair is given by

(1) $$\begin{array}{cc} U=\sum_{i=1}^{\nu }X_{i}, & V=\sum_{i=1}^{\nu }Y_{i}, \end{array}$$

where *X*_*i *_and *Y*_*i *_are two sequences of independent and identically distributed (i.i.d.) random variables (r.v.s), while *X*_*i *_and *Y*_*i *_in each pair (*X*_*i*_, *Y*_*i*_) may be dependent with joint distribution function *F*(*x*, *y*). Limiting themselves to the case where *ν *is non-random, Chu et al. [1] showed that, except for some very special and biologically irrelevant cases, the Markov factorization admitted by the expression levels within individual cells does not survive the summation (aggregation) in formula (1), thereby stymieing any network inference based on the joint distribution. The importance of this observation cannot be emphasized enough. However, as apparent from the relevant literature, it went entirely unnoticed.

In their concluding remarks, Chu et al. [1] note that the mean vector and covariance matrix remain "invariant under aggregation up to a simple linear transformation". The same is obviously true for the correlation matrix. They saw some hope in that fact as reflected in the following quote from their paper: "Thus, while waiting for the technologies capable of measuring efficiently the expression levels in single cells, in experimental studies, we can still make valid – although probably more limited – inferences about the regulatory networks based only on the first two moments of the joint distribution and the independence relations."

Unfortunately, this hope is deflated when considering the case of random *ν*. Indeed, let each *X*_*i *_have the same distribution as *X*, while each *Y*_*i *_is distributed as *Y*. Then the following formula holds for the correlation coefficient *ρ*(*U*, *V*) between *U *and *V *:

(2) $$\rho (U,V)=\frac{\mu_{\nu }Cov(X,Y)+\sigma_{\nu }^{2}\mu_{x}\mu_{y}}{\sqrt{\mu_{\nu }\sigma_{x}^{2}+\sigma_{\nu }^{2}\mu_{x}^{2}}\sqrt{\mu_{\nu }\sigma_{y}^{2}+\sigma_{\nu }^{2}\mu_{y}^{2}}},$$

where *μ*_*ν *_= E(*ν*), *μ*_*x *_= E(*X*), *μ*_*y *_= E(*Y*), $\sigma_{\nu }^{2}$ = Var(*ν*), $\sigma_{x}^{2}$ = Var(*X*), $\sigma_{y}^{2}$ = Var(*Y*), and Cov(*X*, *Y*) is the covariance between *X *and *Y*. Formula (2) can be represented as

(3) $$\begin{array}{l} \rho (U,V)=\frac{\rho (X,Y)}{\sqrt{1+a^{2}\tau }}\frac{1}{\sqrt{1+b^{2}\tau }}\\ +\frac{\tau ab}{\sqrt{1+a^{2}\tau }\sqrt{1+b^{2}\tau }}, \end{array}$$

where *τ *= $\sigma_{\nu }^{2}$/*μ*_*ν*_, *a *= *μ*_*x*_/*σ*_*x*_, *b *= *μ*_*y*_/*σ*_*y*_, and *r *= *ρ*(*X*, *Y*) is the coefficient of correlation between *X *and *Y*. Therefore, *ρ*(*U*, *V*) = *ρ*(*X*, *Y*) if and only if *σ*_*ν *_= 0.

*Remark 1*. If the hybridization reaction reaches equilibrium, an assumption widely adopted in the physical chemistry of microarrays [2], the random variable (r.v.) *ν *can be interpreted as the total number, *N*, of cells from which the total RNA is extracted. In the practical use of microarray technology, however, the reaction is typically stopped before equilibrium has been reached. In the latter case, the r.v. *ν *represents the number of cells that collectively yield the ultimate number of bound target-probe duplexes. Therefore, the random parameter *ν *is unobservable and should be thought of as a virtual number of cells associated with each batch of target RNA produced by them. This notion provides a constructive way of bridging the processes of gene expression at the genomic and tissue levels, which is the main thrust of our discussion. The conventional protocol of a microarray experiment implies that it is the total amount of RNA that is controlled (kept constant) across the arrays (subjects) rather than the number of cells ending up on each array. Therefore, the random fluctuations of *ν *cannot be controlled directly. Even if a tight control of *N *could be provided in experiments, it is unclear whether this would have had a diminishing effect on the variance of *ν*.

An upper bound for the deviation between *ρ*(*U*, *V*) and *ρ*(*X*, *Y*) is given by

(4) $$|\rho (U,V)-\rho (X,Y)|\leq \frac{1}{2}\tau \left({\left(a+b\right)}^{2}+a^{2}b^{2}\tau \right).$$

This result follows from formula (3) and the following chain of inequalities:

$$\begin{array}{l} \left|\rho (U,V)-\rho (X,Y)\right|\leq \\ |\rho (X,Y)\left(1-\frac{1}{\sqrt{1+a^{2}\tau }\sqrt{1+b^{2}\tau }}\right)-\\ \frac{\tau ab}{\sqrt{1+a^{2}\tau }\sqrt{1+b^{2}\tau }}|\\ \leq 1-\frac{1}{\sqrt{1+a^{2}\tau }\sqrt{1+b^{2}\tau }}\\ +\tau ab\leq \\ \frac{(a^{2}+b^{2})\tau +a^{2}b^{2}\tau^{2}}{\sqrt{1+a^{2}\tau }\sqrt{1+b^{2}\tau }\left(\sqrt{1+a^{2}\tau }\sqrt{1+b^{2}\tau +1}\right)}\\ +\tau ab\leq \frac{1}{2}\left((a^{2}+b^{2})\tau +a^{2}b^{2}\tau^{2}\right)\\ +\tau ab=\frac{1}{2}\tau \left({\left(a+b\right)}^{2}+a^{2}b^{2}\tau \right). \end{array}$$

Recall that the equality *ρ*(*U*, *V*) = *ρ*(*X*, *Y*) holds when *τ *= 0. Considering *R *= *ρ*(*U*, *V*) as a function of *τ*, one can verify that *R*(*τ*) either increases monotonically or attains a minimum before starting to increase with increasing *τ*. In both cases, *R *→ 1 when *τ *→ ∞. The function *R*(*τ*) is smooth at *τ *= 0, but its initial slope may be quite high as our sample computations show. An additional quantitative insight into the potential impact of this unobservable variation on the correlation structure of microarray data is possible as described in Section 4.

## 3. An alternative representation of *ρ*(*X*, *Y*) and its implications

Recalling the model given by (1), we give a formula that allows us to better understand the principal difficulty brought about by the random nature of the parameter *ν*. In the [Additional file 1] we find the correlation between the unobservable r.v.s $\frac{1}{\nu }$*U *and $\frac{1}{\nu }$*V*:

(5) $$\rho \left(\frac{1}{\nu }\sum_{i=1}^{\nu }X_{i},\frac{1}{\nu }\sum_{i=1}^{\nu }Y_{i}\right)=\rho (X,Y).$$

This formula implies that estimating the correlation between the unobservable variables *X *and *Y *in each gene pair amounts to estimating the correlation between their averages over a random number of cells, thereby showing the earlier-mentioned nonidentifiability aspect of the problem in terms of the basic random variables. Note that the model given by (1) can be represented as

$$\begin{array}{c} U=\nu \left(\frac{1}{\nu }\sum_{i=1}^{\nu }X_{i}\right)=\nu \bar{X},\\ V=\nu \left(\frac{1}{\nu }\sum_{i=1}^{\nu }Y_{i}\right)=\nu \bar{Y}, \end{array}$$

where the correlation between $\bar{X}$ and $\bar{Y}$ is the same as that between *X *and *Y*, albeit the distributions of the corresponding vectors can be arbitrarily dissimilar. The above representation shows that the r.v. *ν *can be interpreted as a multiplicative random noise as long as the main focus is on pairwise correlations. However, this interpreation is to no avail. The noise *ν *and the signals $\bar{X}$ and $\bar{Y}$ are inherently dependent under this model. Therefore, the popular model of independent random effect is unlikely to serve a good approximation to the aggregated signals. In Section 6, we will invoke formula (5) in our discussion of the utility of the Law of Large Numbers within the framework of model (1).

Formula (5) also illustrates one restrictive assumption behind the model that may have gone unnoticed in its construction. Specifically, the assumption that (*X*_*i*_, *Y*_*i*_) are i.i.d. random vectors implies exchangeability of these vectors across cells and subjects so that the joint distribution of (*X*, *Y*) exhaustively describes both types of variability in formula (1). Put another way, the baseline joint distribution of expression levels of all genes introduced at the cellular level is implicitly compounded with respect to a latent random parameter describing the inter-subject variability. In this case, the correlation between expression signals within each cell appears to be the same as the correlation between their random averages (as formula (5) shows), both correlations being computed across subjects. If one wants to separate the two types of biological variability in a mechanistic model, e.g., by incorporating a random effect into the expression signals associated with single cells and thus making them dependent within each subject, the resultant formulas will become quite cumbersome and contain additional unobservable parameters.

## 4. Assessing the effect of signal aggregation

While our discussion at the end of the previous section suggests that model (1) is quite simplistic, we presently have no better vehicle to assess the potential deviation of the correlation between *X *and *Y *from that between *U *and *V*. To gain an idea of how strong the effect of the parameter *ν *variability can be, let us first compute the coefficient *R *= *ρ*(*U*, *V*) for some parameter values, assuming that gene expressions within single cells are stochastically independent (*ρ*(*X*, *Y*) = 0). By way of example, suppose *σ*_*ν*_/*μ*_*ν *_= 0.23 and *μ*_*ν *_= 2 × 10^5 ^cells. From formula (3), we obtain *R *= 0.999942 for *a *= 1, *b *= 2 and *R *= 0.999952 for *a *= 1, *b *= 5. When setting *ρ*(*X*, *Y*) = 0.5 or *ρ*(*X*, *Y*) = 0.9, the values of *R *change only in the fifth digit. The same magnitude of *R *still stands for *ρ*(*X*, *Y*) = 0 and even when *ρ*(*X*, *Y*) = -0.9. Notwithstanding arbitrariness of the chosen parameters, this indicates an extremely serious problem arising in studies of dependence structures in general and regulatory networks in particular.

Do our calculations imply that the true correlations between gene expressions are absent or weak? The answer is definitely "No" for the following three reasons. First, the assumption of gene independence is biologically implausible and in conflict with a large body of independent experimental evidence, including the known effects of noncoding RNAs and involvement of genes in biochemical pathways. Second, the situation observed in real data is not as severe as in our sample computations: positive correlations tend to be lower and even a small proportion of negative correlations has been documented. It would appear reasonable that many strong negative correlations are hidden in the overwhelmingly positive correlation structure of microarray data. Third, the unobservable parameters chosen in our computations may be very far from reality. Therefore, we have to base our assessment on real gene expression data rather than imaginary parameters of the model. One possible approach to real data analysis is presented below.

*Remark 2*. It should be noted that negative correlations are typically much more prevalent in normalized versus not normalized data. This does not mean, however, that the commonly used normalization procedures can restore the true correlations. A profound effect of such procedures on the correlation structure of microarray data is well-documented [3,4]. This effect is hardly beneficial as normalization procedures distort the aggregated signals in an unpredictable way [5] and interfere in the true correlation structure [3]. There are also other theoretical reasons for the fact that data normalization does not provide a satisfactory solution to the problem; these reasons will be discussed at length in another paper.

From formula (2) it follows that

(6) $$\rho (X,Y)=\frac{\rho (U,V)\sigma_{u}\sigma_{v}-z_{\nu }^{2}\mu_{u}\mu_{v}}{\sqrt{\left(\sigma_{u}^{2}-z_{v}^{2}\mu_{u}^{2})(\sigma_{v}^{2}-z_{v}^{2}\mu_{v}^{2}\right)}},$$

where *z*_*ν *_= *σ*_*ν *_/*μ*_*ν*_. As a function of *z*, the coefficient *ρ*(*X*, *Y*) either decreases monotonically or attains a maximum at the point

(7) $$z^{*}=\frac{\sqrt{2ab-a^{2}R-b^{2}R}}{\sqrt{a^{3}b+ab^{3}-2a^{2}b^{2}R}},$$

where

$$\begin{array}{ccc} R=\rho (U,V), & a=\frac{\mu_{u}}{\sigma_{u}}, & b=\frac{\mu_{v}}{\sigma_{v}}. \end{array}$$

Therefore, the effect of signal aggregation is not unidirectional – the correlation coefficient *ρ*(*X*, *Y*) may be smaller or higher than the observed coefficient *ρ*(*U*, *V*). Formula (6) can be represented in a more concise form

(8) $$\rho (X,Y)=\frac{\rho (U,V)\xi_{u}\xi_{v}-z_{v}^{2}}{\sqrt{\left(\xi_{u}^{2}-z_{v}^{2})(\xi_{v}^{2}-z_{v}^{2}\right)}},$$

where *ξ*_*u *_= *σ*_*u*_/*μ*_*u*_, *ξ*_*v *_= *σ*_*v*_/*μ*_*v *_are the corresponding variation coefficients.

All the parameters entering formulas (6) or (8) can be estimated from microarray data except for *z*_*ν*_, which is unobservable. However, there are natural mathematical constraints that must be imposed on *z*_*ν*_. First of all, we have to require that *z*_*ν *_<*ξ*_*u *_for any gene, i.e.,

(9) $$z_{\nu }<\underset{1\leq j\leq m}{\min}\xi_{u_{j}},$$

where $\xi_{u_{j}}$, *j *= 1,..., *m*, is the variation coefficient for the *j*th gene and *m *is the total number of genes. However, condition (9) does not ensure that |*ρ*(*X*, *Y*)| ≤ 1. To meet the second condition, we derive from (6) the following requirement:

(10) $$z_{\nu }^{2}\leq \frac{\sigma_{u}^{2}\sigma_{v}^{2}[1-\rho^{2}(U,V)]}{\text{Var}(\mu_{u}V-\mu_{v}U)},$$

for all pairs of genes simultaneously.

The above conditions allow us to deduce a realistic range of possible values of the unobservable variation coefficient *z *from a specific set of microarray data. If *ρ*(*X*, *Y*) appears to be a monotonically decreasing function of *z*_*ν*_, which property can be verified with real data, then we can use formula (6) to estimate its maximal deviation from *ρ*(*U*, *V*) by evaluating *ρ*(*X*, *Y*) at the right extreme of *z*_*ν *_yielded by conditions (9) and (10). In this case, we obtain a reasonably realistic upper estimate of the actual effect of signal aggregation in accordance with model (1). If *ρ*(*X*, *Y*) passes through a maximum as a function of *z*_*ν*_, this estimate will become conservative to shifts towards lower values of the true correlation coefficients. Such estimates need to be produced for all gene pairs, of course. More accurate estimates of the effect in both directions (up and down) can be obtained by evaluating the behavior of *ρ*(*X*, *Y*) over the whole range of admissible values of *z*_*ν *_in each gene pair, but this approach is computationally extremely expensive and requires parallel computations.

The mean and minimal (across genes) variation coefficients of gene expression were estimated from the following five sets of microarray data:

BCC: Breast cancer cells cultured *in vitro *(represented solely by "vehicle" control samples that were treated with the medium used to solubilize the inhibitor) with HG U133A Affymetrix Chip used to produce microarray measurements [6];

TELL and HYPERDIP: two types of childhood leukemia, U95A Affymetrix Chip [7];

PCTUM: prostate cancer, U95Av2 Affymetrix Chip [8];

PCNORM: normal prostate tissue obtained from prostate cancer patients, U95Av2 Affymetrix Chip [8].

The results are shown in Table 1. These estimates are consistent with the earlier reported observation that the variation coefficients of gene expression are virtually constant across genes [9]. Using the above-described approach, we analyzed all gene pairs in the HYPERDYP data set reporting expression levels of *m *= 7084 genes for *n *= 88 patients with a specific type of childhood leukemia. In this case, Table 1 offered min $\xi_{u}^{2}$ = 0.044 as an upper bound for $z_{\nu }^{2}$. A more accurate estimate of 0.041 was given by inequality (10). Therefore, we used the latter value as the conservative estimate of $z_{\nu }^{2}$ when computing the correlation coefficient *ρ*(*X*, *Y*) by formula (6). Testing for monotonicity was performed by partitioning the admissible range of $z_{\nu }^{2}$ (given by condition (10)) into four intervals and using formula (6) to compute the corresponding increments of *ρ*(*X*, *Y*) for each interval. If at least one increment happened to be positive in a given pair, this event was recorded as a "monotonicity violation". There were less than 0.2% of all gene pairs that could be suspected for such violations in the HYPERDYP data. While this frequency of monotonicity violation may be reckoned as quite small, it should be kept in mind that possible shifts in *ρ*(*X*, *Y*) towards values higher than the observed *ρ*(*U*, *V*) were entirely ignored in this analysis.

**Table 1:** Variation coefficients of gene expression levels estimated from different data sets.

Dataset	Av *ξ*_*u*_	min *ξ*_*u*_	$\sigma_{\xi_{u}}$	# genes
TELL	0.235	0.188	0.025	7084
HYPERDIP	0.269	0.211	0.029	7084
BCC	0.303	0.175	0.090	10212
PCNORM	0.318	0.213	0.11	7084
PCTUM	0.280	0.152	0.12	7084

Let us now evaluate the numerical results of this study. For the HYPERDIP data set, the mean (over all gene pairs) value of *ρ*(*U*, *V*) is 0.904 and the corresponding standard deviation equals 2.34 × 10^-5^. For the unobservable coefficient *ρ*(*X*, *Y*) these parameters are 0.797 and 4.16 × 10^-5^, respectively. The total number of gene pairs with negative values of *ρ*(*U*, *V*) is only 9442. The number of negative values of *ρ*(*X*, *Y*) is much larger, it equals 223,826 in the data set under study. To gain a better idea of how dissimilar *ρ*(*U*, *V*) and *ρ*(*X*, *Y*) may be, it is worth estimating the mean and standard deviation (across all gene pairs) of the relative deviation

(11) $$\Delta_{\rho }=\left|\frac{\rho (X,Y)-\rho (U,V)}{\rho (U,V)}\right|.$$

The resultant estimates are 0.154 and 8 × 10^-4^, respectively. This does not strike us as a formidable relative difference. However, two caveats are in order here. First, the above estimates are not very stable. If we replace $z_{\nu }^{2}$ = 0.041 with $z_{\nu }^{2}$ = 0.035, the mean value of Δ_*ρ *_falls to 0.112, while the number of gene pairs with negative values of *ρ*(*X*, *Y*) goes down to 109,574. Second, the model (1) user for assessing the deviation Δ_*ρ *_may still be overly simplistic discussed in the previous section. Much more research needs to be done, both theoretically and experimentally, to shed more light on this methodological difficulty.

## 5. Signal aggregation and technical noise

Our estimates in Table 1 and those resulted from condition (10) give only a rough idea of the magnitude of *σ*_*ν*_/*μ*_*ν *_and making them more accurate is highly desirable. We discuss one possibility to attain these ends in the present section. Consider an experimental design that supposedly eliminates the biological variation, thereby yielding the information on measurement errors only. Suppose that a sample of *n *arrays is available that consists solely of technical replicates representing gene expression measurements taken from one and the same subject. Proceeding from the traditional multiplicative noise model,

$$\begin{array}{cc} {\tilde{X}}_{j}=\epsilon_{j}X_{j}, & j=1,...,m, \end{array}$$

where *m *is the total number of genes (probe sets), ${\tilde{X}}_{j}$ is the observed random signal, and *ϵ*_*j *_is an independent random technical (both gene- and array-specific) noise, one would model this situation as

(12) $$\begin{array}{cc} {\tilde{X}}_{j}=\epsilon_{j}C_{j}, & j=1,...,m, \end{array}$$

where *C*_*j *_are nonrandom constants. If the expression levels are log-transformed, we have

$$\log{\tilde{X}}_{j}=\log\epsilon_{j}+\log C_{j}.$$

Therefore,

$$\text{Var}(\log{\tilde{X}}_{j})=\text{Var}(\log\epsilon_{j}),$$

so that, relying on model (12), one can measure the variance, Var(log *ϵ*_*j*_), of the log-transformed technical noise directly from technical replicates. In particular, one can estimate the variance of the mean error across all probe-sets, i.e.,

$$\sigma_{\bar{\epsilon }}^{2}=\text{Var}\left\{\frac{1}{m}\sum_{j=1}^{m}\log\epsilon_{j}\right\}.$$

We resorted to the above line of reasoning in [10] when reanalyzing the Microarray Quality Control Study (MAQC) [11]. For this data set, the estimated $\sigma_{\bar{\epsilon }}$ is equal to 0.09. Since the overwhelming majority of genes have typically much larger (> 0.3) standard deviations of their log-expression signals in biological replicates (different subjects), this level of technical noise can be deemed negligibly small. This estimate also leads us to conclude that the true correlation between the unobservable signals log *X*_*j *_is really strong. Indeed, the contribution of $\text{Var}\{\frac{1}{m}\sum_{j=1}^{m}\log X_{j}\}$ to the variance of log-expressions observed in biological data is much larger than the contribution of $\text{Var}\{\frac{1}{m}\sum_{j=1}^{m}\log\epsilon_{j}\}$ estimated independently from the MAQC data, while a strong correlation between true biological signals (i.e., their values in the absence of measurement errors) is the only explanation for such a discrepancy when the number *m *of genes is very large. This also explains why the Law of Large Numbers (LLN) is not met in microarray data when applied to log-expression levels across genes [12,13].

The situation is no longer the same when we proceed from model (1) in an effort to measure the technical noise stemming from the random nature of the parameter *ν*. For any gene *j*, formula (1) gives

(13) $$\begin{array}{cc} U_{j}=\sum_{i=1}^{\nu }X_{ij}, & j=1,...,m, \end{array}$$

and it is the parameter *ν *that plays the role of the technical noise here. It is clear from (13) that the biological variability cannot be entirely eliminated from gene expression signals even when they are produced by purely technical replicates. Designed to assess the technical variability, the experiment described above may only reduce the variance of the r.v.s *X*_*ij *_by eliminating the inter-subject variability, but there will always be some residual biological variability associated with different cells, i.e. "cell to cell" variability. Under such experimental conditions, we have

(14) $$\begin{array}{cc} {\tilde{U}}_{j}=\sum_{i=1}^{\nu }{\tilde{X}}_{ij}, & j=1,...,m, \end{array}$$

where ${\tilde{X}}_{ij}$ are i.i.d. r.v.s representing the expression levels of the *j*th gene in different cells obtained from the same subject and their common (conditional) variance is expected to be smaller than that of *X*_1*j *_in (13). Formula (14) also suggests that the MAQC data are far from ideal for the purposes of noise assessment because the technical replicates in this study were produced from a mix of many dissimilar tissue sources, this heterogeneity of samples may inflate the variance of ${\tilde{X}}_{ij}$ while it should be kept as low as possible.

To remove the scaling factor *C*_*j *_from the model (12), when deriving the variance of its noise component, we log-transformed the observed expression signals ${\tilde{X}}_{j}$. This trick does not work for model (14) and this significantly complicates the noise assessment. More complications arise when extending the model represented by formula (14) to include an additive term that describes sources of variation other than *ν*. Under the extended model, the original expression level of the *j*th gene in technical replicates is given by

$${\tilde{U}}_{j}=\sum_{i=1}^{\nu }{\tilde{X}}_{ij}+\eta .$$

In the presence of the noise component attributable to *ν*, the error term *η *does not not need to be array-specific as it essentially reflects the equipment-related optical noise.

Since the overall variance of ${\tilde{U}}_{j}$ is expected to be much lower than that of *U*_*j *_[10], one can use technical replicates to make the range of admissible values of *z*_*ν *_(see Section 4) much narrower, thereby providing more accurate estimates of *ρ*(*X*, *Y*) and Δ_*ρ *_in accordance with formulas (8) and (11), respectively. This idea of combining information from biological and technical replicates deserves a careful consideration and even a generous investment in specially-designed experiments because it offers an improved experimental protocol that may make the usual correlation analysis, as well as the network inference based on correlation measures, more meaningful. If the idea works in general, the new protocol will require producing a separate set of technical replicates in each biological experiment in order to estimate the range of *z*_*ν *_and then using this estimate to reconstruct *ρ*(*X*, *Y*) in each gene pair. This suggestion is based on a plausible assumption that the variation coefficient *z*_*ν *_is the same for the biological and technical replicates produced by a given biological experiment. To make the estimate of the range of *z*_*ν *_as accurate as possible, it is imperative that the technical replicates be produced from a homogeneous biological material derived either from one and the same subject or at least from the same type of a tissue (initially collected from several subjects) that is used to produce the corresponding biological replicates. While more laborious and expensive, the experiments thus designed may provide a practically workable solution to the problem discussed in the present paper. Some additional thoughts of this kind are offered in Section 7.

## 6. The law of large numbers and random summation

The following claims seem to be natural in the context of the model given by formulas (1):

1. The observed expression signal *U *is a result of summation of the inter-cellular signals *X*_*i *_over a random number of cells *ν*, thereby defining the basic model structure represented by formulas (1).

The random summands *X*_*i *_are i.i.d. positive r.v.s. independent of *ν*.

2. While the r.v. *ν *is nondegenerate, it tends to take on large values with high probability because the number of cells is expected to be large.

In what follows, we examine some indirect corroborative evidence for the above claims.

Suppose for a moment that the number of summands *ν *= *k *is nonrandom. Then the distribution of the corresponding sum in (1) is *M*-divisible, i.e., it can be represented as the convolution of *M *distribution functions. In this particular case, the fouth central moment *μ*_4_(*U*) satisfies the inequality [14]:

(15) $$\mu_{4}(U)\geq (3-\frac{2}{M})\sigma_{u}^{4}.$$

For infinitely divisible distributions, the condition (15) assumes the form

(16) $$\mu_{4}(U)\geq 3\sigma_{u}^{4}.$$

Under mild conditions, these inequalities hold in the case of random *ν *as well [14]. If the inequality (15) is met in real biological data, this fact will lend additional support to the presence of signal summation in microarray technology. When testing the corresponding inequalities for empirical counterparts of the moments *μ*_4 _(*U*) and *σ*_*u *_in (15) and (16), we observed the event of their violation to be of relatively rare occurrence. For example, the inequality (16) was violated for 18.6% of the 7084 genes in the HYPERDIP data. As expected, this proportion was lower for any finite *M *in (15). Although there is no objective criterion for declaring this frequency consistent with the property of infinite divisibility, we deem it quite low in view of the fact that *μ*_4_(*U*) and *σ*_*u *_in (16) were replaced with their sample counterparts. To corroborate our perception, we generated 7000 independent samples of size *n *= 88 from a log-normal distribution with parameters E(log *U*) = 0.7 and Var(log *U*) = 0.09. The experiment was repeated 1000 times. The mean proportion of "inconsistent" cases was equal to 23.3%, suggesting that the random chance of the event under observation may be high even when the underlying distribution is known to be infinitely divisible.

Yet another underpinning for the presence of signal summation is provided by considering the accompanying distributions of random sums. In the classical summation scheme, the notion of accompanying infinitely divisible distributions was introduced by Gnedenko [15]. This idea was later extended to the random summation by Klebanov and Rachev [16]. Consider the random sum

(17) $$U_{p}=\sum_{i=1}^{\nu_{k}}X_{i},$$

where {*ν*_*k*_, *k *∈ Θ}, Θ ⊂ (1, ∞) is a family of positive integer-valued r.v.s independent of *X*_*i*_, *i *≥ 1. The r.v. The r.v. *ν*_*k *_is assumed to have finite expectation equaling *k *for all *k*. It is known that the random sum *U*_*k *_can be approximated by its accompanying *ν*-infinitely divisible random variable *S*_*k *_under the condition of non-negativity of *X*_*i *_only. Note that the definitions of *ν*-infinitely divisible and accompanying *ν*-infinitely divisible random variables can be found, for exmple, in [17]. In this case, it can be shown [17] that the Laplace transform of *S*_*k *_converges to the Laplace transform of *U*_*k *_in the uniform metric as *k *→ ∞.

Since the number of cells *ν *is expected to be large, it is tempting to apply the Law of Large Numbers (LLN) to the normalized random sum

(18) $$Z_{k}=\frac{1}{\nu_{k}}\sum_{i=1}^{\nu_{k}}X_{i},$$

where *k *is positive integer, and make some predictions based on its behavior as *ν*_*k*_→ ∞ (*k *→ ∞) in probability. As before, we will assume that the sequence of nonnegative integer-valued r.v.s *ν*_*k *_is independent of *X*_*i*_, *i *≥ 1 and *ν*_*k*_→ ∞ (in probability) as *k *→ ∞. The continuous r.v.s *X*_*i *_are i.i.d. and positive. If *μ*_*x *_is finite, it is known [18] that *Z*_*k*_→ *μ*_*x *_as *ν*_*k *_→ ∞, with both limit relations holding in probability as *k *→ ∞. This is the LLN for random sums.

There is no way of ascertaining whether the LLN is met in real microarray data because the r.v. *ν *is unobservable. However, we intend to use this powerful tool to predict certain properties of expression signals and then verify them with real data. In doing so, we rely on the following simple result.

**Assertion 1**. *Under the above conditions, the random vector ***Z**_*k *_= *Z*_1*k*_,..., *Z*_*mk*_, *with its components defined by*

(19) $$\begin{array}{cc} Z_{jk}=\frac{1}{\nu_{k}}\sum_{i=1}^{\nu_{k}}X_{i,j}=\frac{U_{k,j}}{\nu_{k}}, & j=1,...,m, \end{array}$$

*converges in distribution *($\overset{d}{\underset{}{\to }}$) *to a degenerate random vector as k *→ ∞.

The proof is given in [Additional file 2].

The fact that the multivariate limit distribution of **Z**_*k *_is a degenerate one is consistent with the asymptotic behavior of Cov(*U*/*ν*, *V*/*ν*) (and consequently Var(*U*/*ν*), Var(*V*/*ν*)) considered in Section 3. Indeed, we have

(20) $$\text{Cov}\left(\frac{U_{k}}{\nu_{k}},\frac{V_{k}}{\nu_{k}}\right)=E\left\{\frac{1}{\nu_{k}}\right\}\cdot \text{Cov}(X,Y).$$

It is easy to show that $E\left\{\frac{1}{\nu_{k}}\right\}$ → 0 as *k *→ ∞. Therefore, the covariance in (20) tends to zero when *ν*_*k *_is large in probability. The same is true for the variances of *U*_*k*_/*ν*_*k *_and *V*_*k*_/*ν*_*k *_of course. While this behavior of the two central moments is consistent with the convergence established in Assertion 1, the correlation coefficient *ρ *is not well-defined for degenerate random vectors. At the same time, the fact that all components of **Z**_*k *_are asymptotically independent is not in conflict with formula (5) because the latter is valid for any value of *ν*. Nor does it come into conflict with the observation that the intra-cellular gene expression levels are strongly correlated. When deriving formula (5), we divide Cov(*U*_*k*_/*ν*_*k*_, *V*_*k*_/*ν*_*k*_) by the product of the corresponding standard deviations of *U*_*k*_/*ν*_*k *_and *V*_*k*_/*ν*_*k*_, which is why the proportionality coefficient $E\left\{\frac{1}{\nu_{k}}\right\}$ cancels out and the uncertainty manifesting itself in the limit distribution becomes resolved.

The results given above imply that, while the r.v.s *U*/*ν *and *V*/*ν *are asymptotically independent, the correlation between the components of each pair (*X*_*i*_, *Y*_*i*_) may be arbitrarily strong even when *ν *takes on large values with high probability. Such "paradoxical" situtions are not uncommon in the theory of probability. It should be emphasized that the above assertion is valid for the random sum of *ν *i.i.d. random summands normalized by the same random variable *ν*, and not for other possible ways of normalization. For limit theorems related to the random sums normalized by sequences of nonrandom numbers we refer the reader to [19,20].

Now we are in a position to make and test the following two predictions:

*Prediction 1*. The ratios of the observed expression levels *U*_*j *_and *U*_*r*_, *j *≠ *r*, where *j*, *r *= 1,..., *m *and *m *is the total number of genes, tend to have small variances. The covariance between different ratios *U*_*j*_/*U*_*r *_is expected to be small as well.

Indeed, proceeding from the LLN, we expect the asymptotic relation

(21) $$\begin{array}{cc} \frac{U_{j}}{U_{r}}=\frac{\frac{1}{\nu }\sum_{i=1}^{\nu }X_{ij}}{\frac{1}{\nu }\sum_{i=1}^{\nu }X_{ir}}\sim \frac{EX_{1j}}{EX_{1r}}, & j\neq r, \end{array}$$

to hold true as *ν *→ ∞ in probability. This suggests that every ratio *U*_*j*_/*U*_*r *_is virtually constant (across arrays). The above-proven assertion also suggests that every two ratios of the form:*U*_*j*_/*U*_*r *_and *U*_*l*_/*U*_*q *_(with different indices) have small covariances.

**Figure 1:**
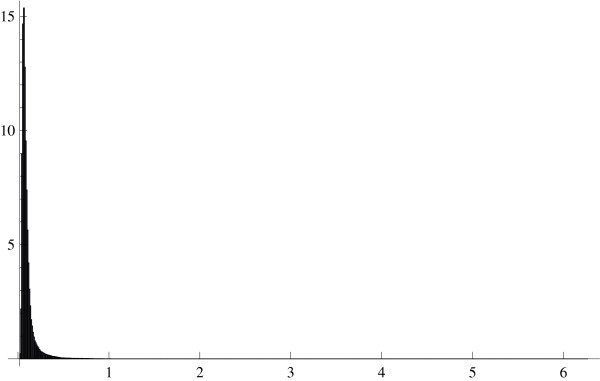
Histogram of standard deviations for the ratios of expression levels in all gene pairs formed from 1000 genes. The HYPERDIP data set.

**Figure 2:**
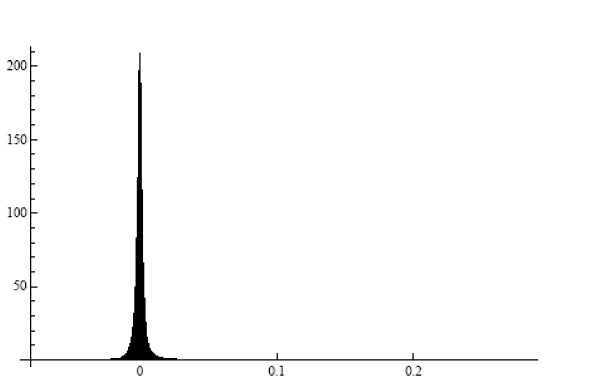
Histogram of covariances between the ratios of gene expressions in all quadruples from a subset of 100 genes. The HYPERDIP data set.

To verify *Prediction 1*, we formed all pairs from 1000 randomly selected genes. The mean (over the gene pairs) standard deviation of the ratios *U*_*j*_/*U*_*r*_(*j *≠ *r*) in the HYPERDIP data was equal to 0.102, which value is very small compared to the corresponding mean of the estimated expectations E(*U*_*j*_/*U*_*r*_), the latter value being equal to 1.044. The histogram of the standard deviations in Figure 1 illustrates this point further. Shown in Figure 2 is the histogram of the estimated covariances between *U*_*j*_/*U*_*r *_and *U*_*l*_/*U*_*q *_in all quadruples formed from 100 randomly selected genes in the HYPERDIP data set. It is clear that they tend to be small as well. This observation explains the most salient properties of the so-called *δ *– *sequence *[12], as well as a remarkable success of significance testing for differential expression of genes when the relevant methods are applied to the elements of this sequence rather than to the original expression levels [12,13].

*Prediction 2*. The average (expectation) of the ratio *U*_*j*_/*U*_*r *_is approximately equal to the ratio of the averages of *U*_*j *_and *U*_*r*_, *j *≠ *r*.

Invoking the LLN, we can assert that

$$\begin{array}{cc} EU_{j}=E\nu EX_{1j}, & EU_{r}=E\nu EX_{1r}. \end{array}$$

Proceeding from the representation

$$\frac{U_{j}}{U_{r}}=\frac{\frac{1}{\nu }\sum_{i=1}^{\nu }X_{ij}}{\frac{1}{\nu }\sum_{i=1}^{\nu }X_{ir}},$$

we can claim that

(22) $$E\left(\frac{U_{j}}{U_{r}}\right)\approx \frac{EU_{j}}{EU_{r}},$$

whenever *ν *is large with high probability.

**Figure 3:**
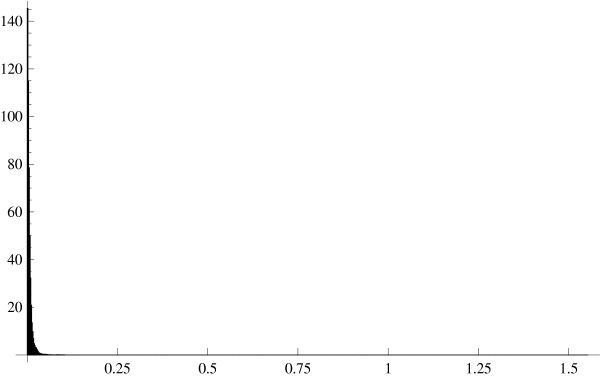
Histogram of the differences between E(*U*_*j*_/*U*_*r*_) and E(*U*_*j*_)/E(*U*_*r*_) estimated by replacing the expected values with the corresponding sample means. The HYPERDIP data set.

Replacing the expected values with their sample counterparts, we computed the absolute difference between the left and right hand sides of the equality (22) for all gene pairs formed from 1000 randomly selected genes in the HYPERDIP data set. The resultant histogram (Figure 3) clearly indicates that such differences are very small with the mean (across the gene pairs) being equal to 0.006.

Hence, both predictions appear to be consistent with real data. Similar analyses of the other data sets referred to in Section 3 have confirmed this conjecture.

## 7. Discussion and concluding remarks

Methods of network reconstruction from designed gene perturbation experiments are beyond the scope of this paper. The fact that the latter strategy can be limited to mean expression levels makes it fundamentally different from the inference based on genome-wide expression measurements. Some limitations of the network inference from gene perturbation experiments have been discussed by other authors (see, e.g., [1]). The multiple testing aspect of the problem will not be touched upon either despite its direct bearing on this type of data analysis.

The world of stochastic phenomena is complex and uncanny. Intuition is not the best guide in that world. Some stochastic effects may seem to defy common sense but nevertheless they may be very real from the theoretical and practical perspectives.

In the present paper, we describe and explore to the best of our ability the impact of random signal aggregation on the correlation structure of microarray gene expression data. While our analysis of real data suggests that this impact may be deemed reasonably moderate in some situations, the main concern still remains because the estimates employed are not sufficiently stable and the underlying model may still need further refinements. A similar concern arises in regard to other standard measures of dependence such as the mutual information. The latter characteristic is applied extensively to the same data structure for the purposes of relevance network inference [21], thereby calling for a similar investigation of its properties.

We overlooked the phenomenon of signal aggregation when discussing the correlation structure of microarray data in our earlier publications [12,9]. The results of [12] connected to the use if *δ*-sequences find now theoretical basis (see Prediction 2). The influence of signal aggregation on Type-A dependency remains not completely clear. In [9], we tried to make the case that the observed strong and long-ranged correlation between gene expression levels are of a biological nature rather than a technical flaw of the microarray technology. Our belief was based on the premise that the effects of the technical noise [10] and multiple targeting [9] on the correlation structure of microarray data appeared to be negligible. There is no reason to revise this premise. However, the effect of random summation of expression signals reported in the present paper is a drastically different story. While technical in nature, this effect represents a serious obstacle standing in the way of correlation analysis and network inference. At the same time, the estimates reported in the present paper still indicate the presence of strong correlations between the expression signals produced by different genes at the level of individual cells.

There are statistical questions, other than the estimation of correlation coefficients, that may be relatively insusceptible to the effect of signal aggregation. For example, we hypothesize that it may still be sensible to compare correlation vectors associated with each gene in two different phenotypes in order to extract more information on pathogenesis of some diseases or responses to drug therapies. However, this conjecture invites a special investigation. It is clear that the crux of the difficulty has to do with a natural desire to make inferences about "microscopic" processes of transcription within individual cells from "macroscopic" observations yielded by gene expression measurements. From this perspective, the mixing effect caused by signal summation should be considered as confounding [22] and, as such, is undesirable. Needless to say, one can employ the correlation coefficients between observed expression signals as more global characteristics of the cell system under study rather than associations between gene activities within each cell. Such characteristics still represent a source of useful information. From this viewpoint, the results of correlation analysis of gene expression data can be interpreted in terms of aggregated (over the cells) genes, an obvious departure from the interpretation that has been in wide use among molecular biologists and bioinformaticians. Since tissue-specific mechanisms regulating cell functions are not well-understood, it is premature to judge whether or not this cautious interpretation is of biological interest.

The most critical question still remains: How can the true correlation be extracted from observed expression levels despite the masking effect of signal aggregation? At present, we have no satisfactory answer to this question. However, some practical expedients mitigating the adverse consequences of signal aggregation can be envisioned. As discussed in Section 5, one approach is to combine the information provided by technical and biological replicates using the mathematical treatment of the problem presented in this paper. Yet another possibility is to modify the experimental protocol so that the total DNA rather than the total RNA be kept constant across the arrays (see Remark 1). The rationale for this suggestion is that the correlation between the parameter *ν *and the total number of cells (gauged by the DNA content) in a given biological sample may well be stronger than that between *ν *and the total RNA. The main problem with hybridization-based technologies is that the latent parameter *ν *is not accessible to direct measurement. The situation is not the same with the sequencing technology that produces counts of all transcripts present in the biological sample. It seems likely that the sequence-based technology offered by Illumina (Solexa) may make it much easier to keep the parameter *ν *constant across biological samples. All the above-mentioned possibilities have yet to be verified in biological experiments, of course.

Finally, a search for measures of dependence or relations between gene expression signals that are preserved under signal aggregation is warranted. For example, introduce the following characteristic

(23) *ρ*(*X*, *Y*) *ξ*_*x*_*ξ*_*y *_= *μ*_*ν *_[*ρ*(*U*, *V*) *ξ*_*u*_*ξ*_*v *_- *ξ*^2 ^(*ν*)].

It is easy to see see, that the inequality

(24) $$\rho (U_{1},V_{1})\xi_{u_{1}}\xi_{v_{1}}>\rho (U_{2},V_{2})\xi_{u_{2}}\xi_{v_{2}}$$

implies

(25) $$\rho (X_{1},Y_{1})\xi_{x_{1}}\xi_{y_{1}}>\rho (X_{2},Y_{2})\xi_{x_{2}}\xi_{y_{2}},$$

because *μ*_*ν *_and *ξ*^2 ^(*ν*) are the same for all genes. Therefore, the coefficient *ρ*(*U*, *V*) *ξ*_*u *_*ξ*_*v *_defined by (23) preserves inequalities between the corresponding coefficients for (*X*, *Y*). In this connection, it is important to recall that the variation coefficient of observed expression levels is almost constant across genes, a fact mentioned in Section 4. Under such conditions, the inequalities (24) and (25) imply the same inequalities for the corresponding correlation coefficients. This suggests that the ranking of gene pairs by the correlation coefficient may still be possible and such inference can probably be improved by stratifying the population of genes by the value of the variation coefficient. Whether this observation is of real utility in studying relationships between genes within the network paradigm has yet to be explored.

The future of the whole research area dealing with regulatory networks hinges on our ability to surmount the obstacle described in the present paper either by means of mathematics (including the recourse to parametric methods) or through radical technological improvements.

## Reviewers' comments

### Reviewer 1 (B.S. Kim)

The authors (K & Y, hereafter) revisited an old issue of statistics, i.e, the problem of aggregation, in a new biotechnology area with a more complicated mode. It is an old issue in statistics that the correlation at the aggregated level may be quite different from the correlation at the individual level. This phenomenon is often referred to as the Simpson's paradox (Simpson, 1951), or the ecological fallacy (Robinson, 1950). Yule and Kendall (1950) also dealt with this issue in Chapter 13. The primary difference of K&Y's approach is that the number of components in the aggregation is regarded as random, because the number of cells in a tissue, a target material on the microarray slide, is not controlled to be fixed under the current technology and hence subject to the random fluctuations. As K&Y indicate, making inference on the genetic regulatory network (GRN) depends heavily on the finding the true correlation on the individual cell level, not on the aggregated level.

This paper deals with one of the basic and fundamental issues in statistics and biology.

#### Minor Points

1. p.2. line 8 from the top. inference based correlation → inference based on correlation

2. p. 5, line 12 from the top. Norvatis Gene Atlas → Norvatis Gene Expression Atlas

3. p.8, lines 1 and 2 from the top: *ρ *→ *ρ*(*X*, *Y*) (three places)

4. p.8 lines 6–8 from the top. Since you considered non zero correlations such as 0.5, 0.9 and -0.9 in the previous lines, your argument of "in conflict with a large body of independent experimental evidence" was not consistent with the previous sentence. Better to delete or modify the first reason.

5. p. 10. lines 15–17 from the top. There is no evidence for supporting the assertion unless you show small values of Var (*ξ*_*n*_) in Table I next to "Average *ξ*_*n*_" column, say.

6. p. 11 lines 8–9 from the bottom. The measurement error is another source of errors in the microarray experiment in addition to technical and biological variations (Churchill, 2002). It is better to distinguish the measurement error from the technical noise here and throughout the manuscript.

7. p. 12 lines 10–12 from the top. It is not clear to me the purpose of this statement. Better q to indicate clearly what authors want to convey by showing that $\sqrt{\text{Var}\{\frac{1}{m}\sum_{j=1}^{m}\log\xi_{j}\}}$ is slightly less than $\frac{1}{m}\sum_{j=1}^{m}\sqrt{\text{Var(}\log\xi_{j})}$.

8. p. 12 lines 8–10 from the bottom. There are several meanings of the word "biological" here and throughout the manuscript. At line 10 from the bottom "biological signals" might reduce the confusion if it is changed into "hybridization signals", because you use "biological replicates" for carrying the inter-subject variation (line 14, p. 12). Also at line 8 from the bottom, I may propose using "real experimental" instead of "biological".

9. p. 13 line 3 from the top. How about "cell to cell variability" instead of "biological variability"?

10. p. 15 line 8 from the bottom. It would be nice if authors provide the definition of " *ν*-infinitely divisible", since it is not found in a standard text book such as Laha and Rohatgi (1979).

11. p. 15 line 2 from the bottom. Provide the definition of "*N*".

12. p. 15 Equations (18), (19). I don't quite follow why authors used two subscripts, k and p, for *ν*.

13. p. 16 Equation (20). How about keeping the consistency of notation by changing as follow?

$$\begin{array}{cc} X_{i}^{\left(j\right)}\to X_{i,j}, & U_{k}^{j}\to U_{k,j} \end{array}$$

14. p. 19 lines 1–3 from the bottom. Would you add some discussions on how the result of this paper might affect the results of Klebanov and Yakovlev (2007)?

#### Referfences

Churchill GA. (2002). Fundamentals of experimental design for cDNA microarrays. Nat. Genet. 22:490–495.

Klebanov L, Yakovlev A. (2007). Diverse correlation structures in microarray gene expression data and their utility in improving statistical inference. Annals of Appl. Statist. 1:538–559.

Laha RG, Rohatgi VK (1979). Probability Theory, New York: Wiley.

Robinson WS. (1950). Ecological correlations and the behavior of individuals. American Sociological Review 15:351–357.

Simpson EH. (1951). The interpretation of interaction in contingency table. J. Royal Statist. Soc. B 13:238–241.

Yule GU, Kendal MG. (1950). An introduction to the theory of statistics. New York: Harper. The authors' responses are provided in the text.

### Reviewer 2 (J. Kowalski)

#### General comments

This manuscript proposes an analytical approach that recognizes what the authors refer to as a 'nitty-gritty' aspect of microarray technology in which intra-cellular expression is produced by signals that are in fact aggregated over a random number of individual cells. The authors consider the effect of random signal aggregation on correlation and network inference, two of the most popular analyses tools for microarray analyses. In general, the authors introduce a statistically sound approach to addressing a biological underpinning of microarray technology that has either been overlooked or not widely known but nonetheless important to consider in related analyses. The authors show the important implications of the 'nitty gritty' microarray aspect for inference, particularly with regard to correlation analyses. A revision that addresses the specific comments below would help to better streamline the significance of the work and reinforce the authors' important contribution to analyses of data from genomic association studies.

#### Specific Comments

1) *Abstract*. In the conclusion section it is stated, "while our preliminary analyses suggests that in reality the reported effect may not be as extreme as theoretical considerations allow:". I would suggest a re-wording to the effect that it is important to recognize the source of signal in microarray technology and to theoretically account for it in any related analyses. Whether the effect is extreme or not, the important point, is to recognize and incorporate such signal source for proper inference. I may also try to include the 'nitty-gritty' in the title within the abstract for contextual meaning of the phrase. One suggestion may be in the Background section, "Contrary to this common belief, modern microarray technology produces signals aggregated over a random number of individual cells, a 'nitty-gritty' aspect of such arrays, thereby causing..."

2) *Introduction*. Starting at Line 11, "Methods of network reconstruction..." I would suggest to move this section to the discussion and prefer to see more of the overview of the approach of the authors in the introduction. These latter points focus on what was not done and why as opposed to what was done and why. One suggestion would be to include the beginning part of section 2 as part of the introduction, explaining the observation noted by Chu et al., as section 2 is a bit lengthy in its current form.

3) *Aggregated Expression Intensities*. In remark 2, the discussion about heterogeneous experiments and PCC, thought important, in its current form, appears a bit disjoint from the rest of the text and perhaps could be either greatly shortened or removed.

4) *Equations*. The number of models/formula within the manuscript severely detracts at time from the equally important biological content. One suggestion may be to devise an appendix for some formula and calculations presented. Of note, I did not check the formula and assumed that they were correct.

#### Minor Comments

1) p.2. section 2, line 3:may consider removing the word 'adequately' since it assumes that technology in its current form does reflect intra-cellular signal but is deficient.

2) Table 1. it may be useful to include the number of genes examined in each dataset to obtain estimates.

The authors' responses are provided in the text.

### Reviewer 3 (G. McLachlan)

This paper provides a theoretical account of signal aggregation on the correlation between the measured expression levels between pairs of genes.

The approach and the results derived are quite novel and I recommend its publication in the Journal.

**Authors: **Thank you so much!

## Competing interests

The authors declare that they have no competing interests.

## Authors' contributions

Authors contribute equally to the manuscript

## Supplementary Material

Additional file 1 Appendix 1. Derivation of the formula (5). Mathematical derivation of the formula (5). Click here for file

Additional file 2 Appendix 2. Proof of the Assertion 1. Mathematical proof of the Assertion 1. Click here for file
