# Supplementary material for: A nitty-gritty aspect of correlation and network inference from gene expression data
Source: Biol Direct. 2008 Aug 20;3:35. doi: 10.1186/1745-6150-3-35 (PMC2569917; doi:10.1186/1745-6150-3-35)
Supplement: Additional file 1 — Appendix 1. Derivation of the formula (5). Mathematical derivation of the formula (5). [file 1745-6150-3-35-S1.pdf]

Appendix 1. Derivation of the formula (5)

We have

$$\mathrm{Cov}\left(\frac{1}{\nu}U, \frac{1}{\nu}V\right) =$$

$$\mathbb{E}\Big(\frac{1}{\nu}\sum_{s=1}^{\nu} X_s \frac{1}{\nu}\sum_{k=1}^{\nu} Y_k\Big) - \mathbb{E}\frac{1}{\nu}\sum_{s=1}^{\nu} X_s \mathbb{E}\frac{1}{\nu}\sum_{k=1}^{\nu} Y_k$$

$$= \sum_{n=1}^{\infty}\frac{1}{n^2}\sum_{s=1}^{n}\sum_{k=1}^{n}\mathbb{E}\big(X_s Y_k\big)\mathbb{P}\{\nu = n\} -$$

$$\sum_{n=1}^{\infty}\frac{1}{n}\sum_{s=1}^{n}\mathbb{E}X_s\mathbb{P}\{\nu = n\} \tag{1}$$

$$\sum_{n=1}^{\infty}\frac{1}{n}\sum_{k=1}^{n}\mathbb{E}Y_k\mathbb{P}\{\nu = n\},$$

where $\mathbb{P}$ and $\mathbb{E}$ are the symbols of probability and expectation, respectively. Consider the first term

$$\sum_{n=1}^{\infty}\frac{1}{n^2}\sum_{s=1}^{n}\sum_{k=1}^{n}\mathbb{E}\big(X_s Y_k\big)\mathbb{P}\{\nu = n\} =$$

$$\sum_{n=1}^{\infty}\frac{1}{n^2}\sum_{s=1}^{n}\sum_{k=1}^{n}\mathbb{E}X_s\mathbb{E}Y_k\mathbb{P}\{\nu = n\}$$

$$+\sum_{n=1}^{\infty}\frac{1}{n^2}\sum_{s=1}^{n}\mathrm{Cov}(X_s, Y_s)\mathbb{P}\{\nu = n\} =$$

$$\mathbb{E}X\mathbb{E}Y + \mathrm{Cov}(X, Y)\sum_{n=1}^{\infty}\frac{1}{n}\mathbb{P}\{\nu = n\}.$$

For the last to terms in (1), we have

$$\sum_{n=1}^{\infty}\frac{1}{n}\sum_{s=1}^{n}\mathbb{E}X_s\mathbb{P}\{\nu = n\} = \mathbb{E}X,$$

$$\sum_{n=1}^{\infty}\frac{1}{n}\sum_{k=1}^{n}\mathbb{E}Y_k\mathbb{P}\{\nu = n\} = \mathbb{E}Y.$$

It follows from formula (7) that

$$\mathrm{Cov}\left(\frac{1}{\nu}U, \frac{1}{\nu}V\right)$$

$$= C \cdot \mathrm{Cov}(X, Y),$$

where

$$C = \sum_{n=1}^{\infty} \frac{1}{n} \mathbb{P}\{\nu = n\} = \mathbb{E}\left(\frac{1}{\nu}\right)$$

is an unknown constant. Considering the variances of U/ν and V/ν in a similar way, we arrive at the formulae (5)
