# Supplementary material for: A nitty-gritty aspect of correlation and network inference from gene expression data
Source: Biol Direct. 2008 Aug 20;3:35. doi: 10.1186/1745-6150-3-35 (PMC2569917; doi:10.1186/1745-6150-3-35)
Supplement: Additional file 2 — Appendix 2. Proof of the Assertion 1. Mathematical proof of the Assertion 1. [file 1745-6150-3-35-S2.pdf]

Appendix 2. Proof of the Assertion 1

Denote by $f(\mathbf{t}) = f(t_1, \ldots, t_m)$ the multivariate characteristic function (c.f.) of $(X_{1,1}, \ldots, X_{1,m})$. Then the c.f. of $\mathbf{Z}_k = \mathbf{U}_k/\nu_k$, where $\mathbf{U}_k = U_{k,1}, \ldots, U_{k,m}$, is given by

$$\mathbb{E}e^{i(\mathbf{U}/\nu_k, \mathbf{t})} = \sum_{n=1}^{\infty} f^n(t_1/n, \ldots, t_m/n) \mathbb{P}\{\nu_k = n\}, \tag{1}$$

where $\mathbf{t} = (t_1, \ldots, t_m)$. Let $\mathbf{a} = (a_1, \ldots, a_m)$ be the vector of mean values for $(X_{1,1}, \ldots, X_{1,m})$. We have

$$f^n(\mathbf{t}/n) = \big(1 + i(\mathbf{a}, \mathbf{t})\frac{1}{n} + o(\|\mathbf{t}\|/n)\big)^n \to e^{i(\mathbf{a},\mathbf{t})} = \prod_{j=1}^{m} e^{ia_j t_j}, \tag{2}$$

as $n \to \infty$. The convergence in (1) is uniform with respect to $\mathbf{t}$ taking on values from a compact set. It follows from (1) and (2) that

$$\big|\mathbb{E}e^{i(\mathbf{U}/\nu_k, \mathbf{t})} - e^{i(\mathbf{a},\mathbf{t})}\big| = \big|\sum_{n=1}^{\infty} \Big(f^n(t_1/n, \ldots, t_m/n) - e^{i(\mathbf{a},\mathbf{t})}\Big) \mathbb{P}\{\nu_k = n\}\big| \leq \sum_{n=1}^{\infty} \big|f^n(t_1/n, \ldots, t_m/n) - e^{i(\mathbf{a},\mathbf{t})}\big| \mathbb{P}\{\nu_k = n\}. \tag{3}$$

From (2), we can claim that for any $\varepsilon > 0$ there exists such $N_\varepsilon > 1$ independent on k that

$$\big|f^n(\mathbf{t}/n) - e^{i(\mathbf{a},\mathbf{t})}\big| < \varepsilon \tag{4}$$

for all $n > N_\varepsilon$ and all $\mathbf{t}$ from any fixed compact set. Using (3) and (4) one can write

$$\big|\mathbb{E}e^{i(\mathbf{Z}_k, \mathbf{t})} - e^{i(\mathbf{a},\mathbf{t})}\big| \leq 2\sum_{n=1}^{N_\varepsilon} \mathbb{P}\{\nu_k = n\} + \varepsilon. \tag{5}$$

Since $\nu_k \to \infty$ as $k \to \infty$, we can assert that $\mathbb{P}\{\nu_k = n\} \to 0$ as $k \to \infty$ for any $n \leq N_\varepsilon$. It finally follows from inequality (5) that

$$\big|\mathbb{E}e^{i(\mathbf{Z}_k, \mathbf{t})} - e^{i(\mathbf{a},\mathbf{t})}\big| \to 0 \tag{6}$$

as $k \to \infty$. This completes the proof of the convergence

$$\mathbf{Z}_k \xrightarrow{d} \mathbf{a}. \tag{7}$$
